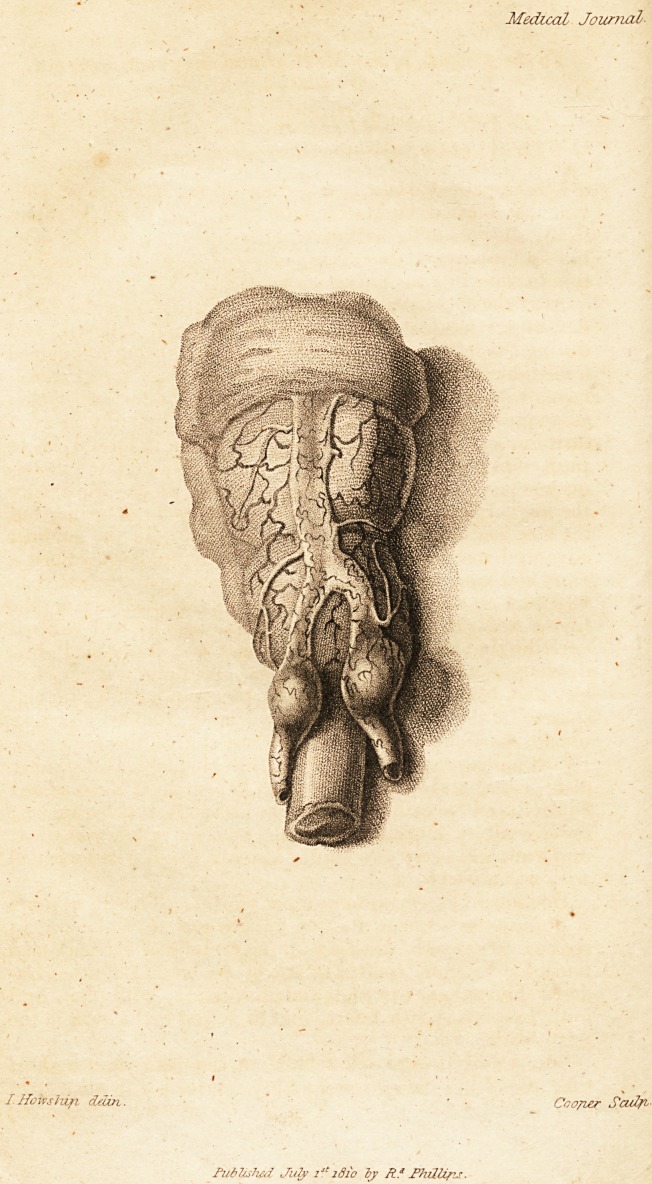# Conclusion of Benj. Brierley's Case

**Published:** 1810-12

**Authors:** John Kenworthy

**Affiliations:** Dobcross, Yorkshire


### *To the Editors of the Medical and Physical Journal.*

### *Conclusion of Benj. Brierley's Case (with a Plate.)*

Gentlemen,

As the patient whose case I drew up for insertion in your valuable Journal in March 1809 (Vol. xxi. p. 225) is since dead, the remaining narrative of his sufferings, and the morbid appearances on dissection, may, perhaps, be a gratification to some of your numerous readers.

After the accidental discharge of hydatids from the bladder of my patient, his miseries were much relieved, and during the following months of winter and spring he enjoyed a tolerable state of health, partaking of some social enjoyments with a relish that he had once thought for ever gone; subsequently he was often distressed with a difficulty of micturition, and was sometimes obliged to strain so forcibly as to push down the rectum very considerably, and the consequence was hæmorrhage. There was not at any time since the discharge of hydatids, a total suppression of urine, but the flow was often not more than guttatim. As the summer advanced and winter approached, his symptoms assumed a more and more serious aspect, until I was again requested to visit him on the 6th of December, sixteen months from the time I first saw him. During several days he had suffered exceedingly; his efforts to make water had occasioned great discharges of blood from the rectum; his pulse was very feeble, and beat about 90 strokes in the minute; hiccup and spasmodic affections throughout the body troubled him very much; particularly affecting the muscles of the abdomen. By examination with my hand, the bladder was distinctly discernable, and seemed as though it contained some urine: I introduced the catheter, and drew away about three ounces; ordered him a draught with emuls. olei. amygdal. and tinct. opii gutt. xv. every two hours, also his belly to be fomented with warm water.

December 7th. Symptoms more violent. There is at times a profuse hæmorhage during an attempt to make water; spasms very severe, drawing up the muscles of the abdomen into a hard tumor, nearly the size of an infant's head. Ordered him æther, camphor and assafœtida, with tinct. opii gutt. xxv. every two hours, and to be put to the neck in the warm bath.

8th. Enjoyed some relief while in the bath, and a short time after; but the spasms speedily returned with very great

 violence; violence; he has had no sleep, pulse feeble and irregular; voids his urine involuntarily in very small quantities: continue his medicine with gutt. xxx. tinct. opii and the warm bath as before.

9th. This day I could not visit him, being detained by a case of midwifery; he therefore continued the use of his antispasmodics.

10th. Spasms, if possible, still more violent, agitating the whole body with great severity: pulse very feeble, fluttering, and intermitting: discharge of blood from the rectum almost continual; his feet cold. Continued his antispasmodic medicine, with tinct. opii gutt. xxxvi.

11th. Died this morning about two o'clock.

13th. By permission of his friends, I proceeded to open the body, a medical friend being present, together with Mr. B. before mentioned (Vol. xxi. p. 226). On laying open the abdomen, the viscera had a healthy appearance; there was a considerable accumulation of fat surrounding the bladder and kidneys; the rectum was somewhat thickened, and the blood-vessels ramifying on it charged with blood. I dissected away the bladder, making the incision to include the prostate gland, (Plate *a*) which was much enlarged. I then laid it on a table, and opened it from the neck to the fundus, which displayed at once the ragged remains of the sac, *(b)* which contained the hydatids, pendulously attached about two inches from the neck of the bladder, the coats of which *(c)* were thickened to about an inch and one-eighth; the inner surface was sacculous, *(d)* and contained about two ounces of a brownish-coloured, thick gelatinous fluid. I next proceeded to take away the kidneys, and opened one of them longitudinally; its pelvis was so much enlarged as to admit a moderate sized hen's egg; the ureter as it departed immediately from the kidney was proportionally wide, and further on its approach towards the bladder was sufficiently capacious to admit my little finger within it. Mr. B. will doubtless feel the importance of this case, pointing out the great necessity of weighing in his mind with much deliberation the obscure symptoms of disease which do sometimes occur, and of investigating cautiously so as to be decidedly confident of the real nature of the complaint. Lamentable indeed must the conclusion of the present case have been, had the operation of lithotomy been performed, and truly degrading to the present enlightened state of medical science!

JOHN KENWORTHY.

*Dobcross, Yorkshire, October* 16, 1810.

## Figures and Tables

**Figure f1:**